# Cutting and Interfering in Horses

**Published:** 1880-07

**Authors:** Daniel E. Dowd

**Affiliations:** Horseshoer, 160 East Fifty-second Street; New York


### ART. XVI.—CUTTING OR INTERFERING IN HORSES —HOW REMEDIED.

BY DANIEL E. DOWD,
HORSESHOER, 160 EAST FIFIY-SECOND SIREET.

HORSES which have never been shod are rarely subject to cutting or interfering. This habit, or, more properly, this fault, has many causes, the principal of which are as follows: Deformity of limbs, which may be either congenital or acquired—that is, caused by debility or disease, by injury or improper shoeing. All of these various conditions, either of which may, in a given case, be the cause of the defect, must be taken into consideration by the practical horse-shoer, with special attention to the last-named. This act in the movement of the animal may take place either in front or behind; it may be either double or single, or it may occur all around. When it occurs in front, the knees, or upper portion of the cannon bone, and occasionally the fetlock joints, are the parts most subject to injury. Behind, only the fetlock joint is involved.

After taking a view of the situation and conditions, the first thing to be done by the shoer, a necessary preliminary, is to ascertain, as near as may be, what part of the striking hoof is engaged. This can readily be accomplished by covering the entire shell on the inside with chalk, and then starting the animal in motion. The part of the hoof which touches will be shown by the chalk being rubbed off by contact with the other leg.

If the feet and legs are in a normal condition, our first effort should be directed to placing on the feet, with perfect evenness, shoes without corks, so that the animal may stand, as near as may be, on a level, and natural plain. If the shells be too much spread, and this is, therefore, where the fault lies, it would then be well to set the shoes a little outward, and rasp oft the inside shell as much as the quarters will allow. Should a nail clinch be a means of injury, place it outside of the striking line.

Should this not overcome the trouble, means to increase the width in stepping must be resorted to. This is accomplished by the following devices: When the cutting takes place at the knees and upper part of the cannon bone, efforts must be directed towards lessening the knee action. This is best done by shoeing as lightly as possible, at the same time paring away the inside of the shell, leaving the outside high, and weighting the shoe on the outside, by making it thick there and thin on the inside. This process, of course, must not be carried to such an extent as to cause injury to the lateral ligaments of the fetlock joint, by straining them; but only so as to make the animal stand and move easily. It is, however, hardly necessary to thus caution any practical horse-shoer of fair abilities.

If the cutting be only of the fetlock joints, a simple weighting of the shoes on the fore feet will usually remedy it.

Cutting behind is commonly on the fetlock joints. The measures for remedying this are just the reverse of those taken for the fore-feet. In this case we thicken up and weight the inside, while paring away the outside, preserving, however, as far as possible, the natural plane of standing.

---

This journal is published in the interests of comparative science, and its columns are open for contributions of original articles, reports of societies, histories of cases of veterinary practice, news items, etc., from all parties interested in the cause. Papers designed to appear in the next issue should reach us by September 15th, at which time the ARCHIVES goes to press. All communications should be addressed

W. L. HYDE & CO.,

22 Union Square, New York.

